# Lifestyle predictors of depression and anxiety during COVID-19: a machine learning approach

**DOI:** 10.47626/2237-6089-2021-0365

**Published:** 2022-04-26

**Authors:** Mario Simjanoski, Pedro L. Ballester, Jurema Corrêa da Mota, Raquel B. De Boni, Vicent Balanzá-Martínez, Beatriz Atienza-Carbonell, Francisco I. Bastos, Benicio N. Frey, Luciano Minuzzi, Taiane de Azevedo Cardoso, Flavio Kapczinski

**Affiliations:** 1 Neuroscience Graduate Program McMaster University Hamilton ON Canada Neuroscience Graduate Program, McMaster University, Hamilton, ON, Canada.; 2 Department of Psychiatry and Behavioural Neurosciences McMaster University Hamilton ON Canada Department of Psychiatry and Behavioural Neurosciences, McMaster University, Hamilton, ON, Canada.; 3 Instituto de Comunicação e Informação Científica e Tecnológica em Saúde Fundação Oswaldo Cruz Rio de Janeiro RJ Brazil Instituto de Comunicação e Informação Científica e Tecnológica em Saúde (ICICT), Fundação Oswaldo Cruz (FIOCRUZ), Rio de Janeiro, RJ, Brazil.; 4 Teaching Unit of Psychiatry and Psychological Medicine Department of Medicine University of Valencia Valencia Spain Teaching Unit of Psychiatry and Psychological Medicine, Department of Medicine, University of Valencia, CIBERSAM, Valencia, Spain.; 5 Department of Medicine University of Valencia Valencia Spain Department of Medicine, University of Valencia, Valencia, Spain.

**Keywords:** Mental health, SARS-CoV-2, lifestyle, machine learning, pandemic

## Abstract

**Introduction:**

Recent research has suggested an increase in the global prevalence of psychiatric symptoms during the COVID-19 pandemic. This study aimed to assess whether lifestyle behaviors can predict the presence of depression and anxiety in the Brazilian general population, using a model developed in Spain.

**Methods:**

A web survey was conducted during April-May 2020, which included the Short Multidimensional Inventory Lifestyle Evaluation (SMILE) scale, assessing lifestyle behaviors during the COVID-19 pandemic. Depression and anxiety were examined using the PHQ-2 and the GAD-7, respectively. Elastic net, random forest, and gradient tree boosting were used to develop predictive models. Each technique used a subset of the Spanish sample to train the models, which were then tested internally (vs. the remainder of the Spanish sample) and externally (vs. the full Brazilian sample), evaluating their effectiveness.

**Results:**

The study sample included 22,562 individuals (19,069 from Brazil, and 3,493 from Spain). The models developed performed similarly and were equally effective in predicting depression and anxiety in both tests, with internal test AUC-ROC values of 0.85 (depression) and 0.86 (anxiety), and external test AUC-ROC values of 0.85 (depression) and 0.84 (anxiety). Meaning of life was the strongest predictor of depression, while sleep quality was the strongest predictor of anxiety during the COVID-19 epidemic.

**Conclusions:**

Specific lifestyle behaviors during the early COVID-19 epidemic successfully predicted the presence of depression and anxiety in a large Brazilian sample using machine learning models developed on a Spanish sample. Targeted interventions focused on promoting healthier lifestyles are encouraged.

## Introduction

The widespread global COVID-19 crisis continues to affect people in different ways, with many people becoming vulnerable to mental health challenges during the pandemic.^1^ Since the onset of this pandemic, fears arising from uncertainties about their well-being have led to major changes in people’s lifestyles around the world.^2^ The sudden deviation from daily routines has resulted in increased prevalence of psychiatric symptoms relative to before the COVID-19 pandemic.^1^ In particular, online web-surveys have been used to assess symptoms of mental disorders and have found an increase of the prevalence of symptoms of common mental disorders such as depression and anxiety in the general population in many countries, such as China, Italy, and Denmark, among others.^3-5^

Two of the most severely affected countries in the world were Spain and Brazil, where citizens experienced high levels of psychological distress in the early stages of the COVID-19 epidemic.^6,7^ Using online assessment tools, research studies conducted during the initial stages of the pandemic in Spain indicated a high prevalence of depressive symptoms, ranging between 18.7%^8^ and 41%^9^, as well as of anxiety symptoms, ranging between 21.6%^8^ and 25%^9^ among the general population. Similarly, a study in Brazil conducted during the COVID-19 epidemic found anxiety and depression to be the most commonly prevalent psychiatric symptoms in the general population, with a staggering 81.9% of participants indicating symptoms of anxiety, and 68% presenting symptoms of depression.^10^ Furthermore, another Brazilian study found a positive association between psychological symptoms (i.e. depression and anxiety) and social isolation variables (i.e. loneliness, days in isolation, level of concern about the COVID-19 situation in Brazil), suggesting the impact that these challenging routine changes may have had on the mental well-being of people.^11^

The major lifestyle adjustments people have had to make during this pandemic might be considered as risk factors for the appearance of unstable psychological symptoms during the quarantine period.^2^ Unhealthy behaviors, such as poor dietary habits, poor sleep quality, and lack of exercise, to name a few, have been found to contribute to the burden of mental health around the globe.^12,13^ Given the unusual circumstances people across the world have been affected by, it is not uncommon for people to have developed unhealthy behaviors during the quarantine period that may trigger stress-related symptoms of depression and anxiety.^2^

A recent systematic review including studies from various countries suggests a global increase in prevalence of psychiatric symptoms during the COVID-19 pandemic,^1^ with changes in everyday routines potentially playing a major role in this regard. However, it is not clear which specific changes to lifestyle behaviors and daily routines have had the greatest impact in triggering symptoms of depression and anxiety in people in highly affected countries. Further examination of this question is important in order to plan effective strategies to target prevention of mental health issues. A significant component of the present study relies on using machine learning techniques to analyze the importance of different lifestyle variables for predicting depressive and anxiety symptoms. Machine learning has become an efficient and accurate instrumental technique for analyzing big data.^14^ Thus, the aim of the present study was to analyze the predictive effect of different lifestyle behaviors as risk factors for depression and anxiety in the general Spanish and Brazilian populations during the COVID-19 pandemic.

## Methods

Data were used from web surveys conducted from April 15 to May 15, 2020 (Spain) and from April 20 to May 20, 2020 (Brazil), as detailed elsewhere.^15,16^ Briefly, individuals aged 18 years or older, living in Brazil or Spain, and having access to the Internet were recruited via social networks (Facebook, WhatsApp, and Twitter) using a snowball technique and sponsored social network advertisements. Individuals who agreed to participate after reading the information and terms of the study provided electronic informed consent. Subsequently, they answered a 101-question questionnaire covering demographics, COVID-19 experience, lifestyle, self-rated health, and previously diagnosed medical and psychiatric conditions, and including brief screenings for depression, anxiety, and risky drinking.

The study was approved by the Ethics Committee at the Hospital Universitari i Politècnic La Fe in Valencia, Spain, and by the Comissão Nacional de Ética em Pesquisa (CONEP, Brazil –3.968.686).

### Predictors assessed

Sociodemographic predictors included age, sex, employment status, educational level, number of people living in the household, and country of residence (Brazil or Spain). Information related to COVID-19 included questions such as, “Did a health professional formally diagnose you with COVID-19?” and “Have you lost a loved one during the pandemic?”

The main predictors of interest were lifestyle behaviors during the COVID-19 quarantine period. These were assessed using the Short Multidimensional Inventory Lifestyle Evaluation (SMILE) scale, a 43-item, self-rated questionnaire comprising 7 lifestyle domains (Diet and Nutrition, Substance abuse, Physical activity, Stress management, Restorative sleep, Social support, and Environmental exposures) developed for multidimensional 30-day lifestyle assessment.^15^ On the SMILE scale, response options are measured with a 4-point Likert scale (Always, Often, Seldom, Never) and the final score is obtained by summing the scores for all questions (noting that some questions are reverse-scored). The higher the score, the better (healthier) the lifestyle. In addition, self-rated health (SRH) was measured using the question “How would you rate your health in general?”, with response options of “Very good”, “Good”, “Regular”, “Bad”, and “Very bad”, scored from 1 to 5, respectively. For the purpose of the present study, all the items were independently included in the model.

### Outcome

Our main outcome was presence of a positive screening result for depression or anxiety. Current depression and anxiety were assessed using Patient Health Questionnaire-2 (PHQ-2)^17^ for depression, with a cut-off of ≥ 3, and the Generalized Anxiety Disorder 7-item (GAD-7)^18^ scale for anxiety, with a cut-off of ≥ 10. Two dichotomous variables were created, where scores on the two scales that were equal to or above the cut-offs were defined as “Positive Depression” and “Positive Anxiety”.

### Dealing with non-responses

First, all participants that had missing variables, which would prevent us from building the final model, were excluded from the analysis. Secondly, columns containing more than 5% of missing data were removed. Finally, the remaining variables were imputed as follows: (1) for every variable, the mode, in case of categorical, or the mean, in case of continuous data, was computed from the training set; (2) the internal test set was imputed with the previously computed modes and means; (3) the same was done for the external test set (Brazil).

### Statistical analysis

The statistical analyses performed to compare groups in terms of sociodemographic and clinical characteristics were conducted using SPSS 21. Independent variables were described by outcome and compared using chi-square tests and Student’s *t* test for independent samples. All variables, with the exception of SMILE scores, were categorized and analyzed using chi-square tests between the respective outcome groups. For SMILE scores, the samples were compared using Student’s *t* test. All machine learning experiments were conducted using R software (version 4.04), and the *caret* library (version 6.0-86).^19^ The data were analyzed using 3 different machine learning algorithms: elastic net, random forest, and gradient tree boosting (extreme gradient boosting [XGBoost] library). Elastic net is a regularized linear model that penalizes high weights, and thus is focused on generalization of the model for unseen samples.^20^ Random forest is a machine learning algorithm that combines and averages the predictions of multiple decision trees with random subsets of features and instances, resulting in a single predictive model.^21^ XGBoost is a scalable technique that creates a predictive model by efficiently adding new models to correct the errors of existing models (also known as ‘gradient boosting’), until the best possible model is reached.^22^ The dataset was trained and tested with each of the three models separately to examine their performance in comparison to each other. For each model, the data from Brazil were separated from the dataset and were not used until testing time; referred to as the *external test*. Then, the Spain dataset was split into two (75% for a training sample and 25% for an internal test) using class-stratified sampling. The training sample was then used to train the model, according to the training procedures for each machine learning technique. For each model, a grid search with the *caret* default hyperparameters was used to identify the best model in a 10-fold cross-validation procedure. Downsampling of the majority class was used to fix class imbalance. Variables with more than 5% missing data were removed and the remaining variables were imputed by either the mode (for categorical variables) or the mean (for numeric variables). Generalization of the model was then assessed in the internal test sample, and used to generate all performance metrics (e.g., accuracy, sensitivity, and others) for the Spanish sample. Finally, the model was evaluated on every individual from the Brazilian sample without any retraining or fine tuning. Model predictions were also used to create risk quintiles. Participants were sorted by their corresponding predicted probabilities and separated into five groups (20% highest predictions allocated to group 1, 20-40% allocated to group 2, and so on), then, the percentage of participants with presence of the outcome was calculated. This approach provides a broad idea of how predicted probabilities translate into actual probabilities of the outcome in test data (i.e. model calibration).

### Data availability

The data that support the findings of this study are available from the corresponding author upon reasonable request.

## Results

### Sample size and sociodemographic characteristics

The final sample for this study comprised 22,562 individuals, with 19,069 subjects from Brazil and 3,493 subjects from Spain. The sociodemographic characteristics were analyzed separately for each country (Brazil or Spain) and further differentiated based on the current clinical symptoms of the individuals, as evaluated by the PHQ-2 and GAD-7 for depression and anxiety symptoms, respectively. The comparisons were performed within the specific subsets of the sample, leading to 4 main comparisons: depression vs. no depression in Brazil, depression vs. no depression in Spain, anxiety vs. no anxiety in Brazil, and anxiety vs. no anxiety in Spain. The sociodemographic differences are described in Table 1 (depression screening) and Table 2 (anxiety screening).

**Table 1:** Sociodemographic and clinical variables in the Brazilian and Spanish samples, by presence of depression

	N = 22,562	Brazil			Spain		

		Yes (9,816)	No (9,253)	p-value	Yes (728)	No (2,765)	p-value
Sex				< 0.001			< 0.001
Female	15,379	6,930 (70.6)	6,064 (65.5)		537 (73.8)	1,848 (66.8)	
Male	7,183	2,886 (29.4)	3,189 (34.5)		191 (26.2)	917 (33.2)	
Age				< 0.001			< 0.001
18-41	13,687	7,466 (76.1)	4,337 (46.9)		514 (70.6)	1,370 (49.5)	
42 or over	8,875	2,350 (23.9)	4,916 (53.1)		214 (29.4)	1,395 (50.5)	
Educational attainment				< 0.001			< 0.001
Elementary/high school	5,749	2,945 (30.0)	1,601 (17.3)		328 (45.1)	875 (31.6)	
University	11,463	4,988 (50.8)	5,073 (54.8)		258 (35.4)	1,144 (41.4)	
Graduate school	5,349	1,883 (19.2)	2,578 (27.9)		142 (19.5)	746 (27.0)	
People in the household				< 0.001			0.151
1	2,856	1,169 (11.9)	1,335 (14.4)		71 (9.8)	281 (10.2)	
2 or 3	12,976	5,641 (57.6)	5,408 (58.5)		382 (52.5)	1,545 (56.0)	
4 to 9	6,695	2,988 (30.5)	2,499 (27.0)		274 (37.7)	934 (33.8)	
Working				< 0.001			< 0.001
No	8,653	4,043 (41.2)	3,201 (34.6)		389 (53.4)	1,020 (36.9)	
Yes	13,135	5,336 (54.4)	5,900 (63.8)		286 (39.3)	1,613 (58.3)	
Lost job during the pandemic	774	437 (4.5)	152 (1.6)		53 (7.3)	132 (4.8)	
Essential worker (yes)	3,717	1,193 (22.4)	1,626 (27.6)	< 0.001	135 (47.2)	763 (47.3)	0.975
Frontline worker (yes)	1,266	369 (30.9)	443 (27.2)	0.033	73 (54.1)	381 (49.9)	0.375
Studying (yes)	3,508	1,930(47.6)	888 (27.7)	< 0.001	250 (64.1)	440 (42.8)	< 0.001
Self-isolated (yes)	17,388	8,260 (84.8)	7,459 (81.1)	< 0.001	408 (56.7)	1,261 (45.9)	< 0.001
Diagnosed with COVID-19	215	80 (0.8)	69 (0.7)	0.587	18 (2.5)	48 (1.7)	0.196
Lost someone in the pandemic (yes)	1,724	771 (7.9)	617 (6.7)	0.002	60 (8.3)	276 (10.0)	0.161
Chronic disease*	6,958	3,009 (30.9)	3,002 (32.6)	0.009	220 (30.5)	727 (26.5)	0.032
Mental Health Disorder*	7,070	4,386 (46.3)	2,126 (23.2)	< 0.001	224 (32.0)	334 (12.2)	< 0.001
Infectious disease*	762	439 (4.5)	310 (3.4)	< 0.001	4 (0.6)	9 (0.3)	0.373
Depression diagnosis*	3,884	2,672 (28.0)	944 (10.3)	< 0.001	135 (19.1)	133 (4.8)	< 0.001
Anxiety diagnosis*	6,451	4,059 (42.7)	1,885 (20.6)	< 0.001	205 (29.0)	302 (11.0)	< 0.001
Alcohol abuse	19,257	4,811 (48.7)	3,976 (42.4)	< 0.001	234 (32.0)	855 (30.6)	0.469
Self-rated health (good/very good)	16,323	5,953 (60.7)	7,863 (85.0)	< 0.001	363 (50.1)	2,144 (77.4)	< 0.001
SMILE (mean, standard deviation)	15,822	115.6 (12.3)	105.4 (11.6)	< 0.001	114.5 (11.1)	106.2 (10.3)	< 0.001

Data presented as n (%), unless otherwise specified.

* Yes, diagnosed within the last 12 months.

**Table 2:** Sociodemographic and clinical variables in the Brazilian and Spanish samples, by presence of anxiety

	N = 22,355	Brazil			Spain		

		Yes (9,400)	No (9,492)	p-value	Yes (716)	No (2,747)	p-value
Sex				< 0.001			< 0.001
Female	15,192	6,789 (72.2)	6,045 (63.7)		537 (75.0)	1,821 (66.3)	
Male	7,163	2,611 (27.8)	3,447 (36.3)		179 (25.0)	926 (33.7)	
Age				< 0.001			< 0.001
18-41	13,592	6,970 (74.1)	4,750 (50.0)		502 (70.1)	1,370 (49.9)	
42 or over	8,763	2,430 (25.9)	4,742 (50.0)		214 (29.9)	1,377 (50.1)	
Educational attainment				< 0.001			< 0.001
Elementary/High School	5,667	2,674 (28.4)	1,804 (19.0)		295 (41.2)	894 (32.5)	
University	11,365	4,796 (51.0)	5,178 (54.6)		275 (38.4)	1,116 (40.6)	
Graduate School	5,322	1,930 (20.5)	2,509 (26.4)		146 (20.4)	737 (26.8)	
People in the household				< 0.001			< 0.001
1	2,804	992 (10.6)	1,466 (15.6)		50 (7.0)	296 (10.8)	
2 or 3	12,889	5,448 (58.1)	5,528 (58.3)		372 (52.0)	1,541 (56.2)	
4 to 9	6,626	2,945 (31.4)	2,483 (26.2)		294 (41.0)	904 (33.0)	
Working				< 0.001			< 0.001
No	8,520	3,645 (38.8)	3,485 (36.7)		352 (49.2)	1,038 (37.8)	
Yes	13,066	5,355 (57.0)	5,822 (61.3)		313 (43.7)	1,576 (57.4)	
Lost job during the pandemic	769	400 (4.3)	185 (1.9)		51 (7.1)	133 (4.8)	
Essential worker (Yes)	3,692	1,281 (23.9)	1,518 (26.1)	0.009	160 (51.1)	733 (46.5)	0.136
Frontline worker (Yes)	1,257	441 (34.4)	365 (24.0)	< 0.001	88 (55.0)	363 (49.5)	0.209
Studying (Yes)	3,459	1,688 (46.2)	1,087 (31.2)	< 0.001	235 (66.4)	449 (42.9)	< 0.001
Self-isolated (Yes)	17,220	7,810 (83.7)	7,759 (82.2)	0.007	365 (51.6)	1,286 (47.1)	0.033
Diagnosed with COVID-19	212	83 (0.9)	65 (0.7)	0.122	23 (3.2)	41 (1.5)	0.002
Lost someone in the pandemic (Yes)	1,712	782 (8.3)	596 (6.3)	< 0.001	65 (9.2)	269 (9.8)	0.593
Chronic disease*	6,886	2,991 (32.1)	2,956 (31.3)	0.254	225 (31.8)	714 (26.2)	0.003
Mental Health Disorder*	6,987	4,433 (48.8)	2,005 (21.4)	< 0.001	247 (36.0)	302 (11.1)	< 0.001
Infectious disease*	751	420 (4.5)	319 (3.4)	< 0.001	5 (0.7)	7 (0.3)	0.071
Depression diagnosis*	3,836	2,564 (28.0)	1,006 (10.7)	< 0.001	133 (19.3)	133 (4.9)	< 0.001
Anxiety diagnosis*	6,379	4,176 (45.9)	1,702 (18.1)	< 0.001	233 (33.7)	268 (9.8)	< 0.001
Alcohol abuse	19,257	4,670 (48.7)	4,117 (42.6)	< 0.001	218 (30.1)	871 (31.1)	0.602
Self-rated health (good/very good)	16,203	5,710 (60.8)	8,000 (84.3)	< 0.001	376 (52.7)	2,117 (77.2)	< 0.001
SMILE (mean, standard deviation )	15,822	115.0 (12.6)	106.3 (11.9)	< 0.001	114.0 (11.2)	106.4 (10.4)	< 0.001

Data presented as n (%), unless otherwise specified.

* Yes, diagnosed within the last 12 months.

### Model performance

Model performance was assessed using several metrics. Table 3 presents all the metrics for the elastic net, random forest, and XGBoost models, comparing the results when the Brazilian sample is used for testing and when the Spanish sample is used for testing. The 3 models performed very similarly for the internal and external tests. Differences can be perceived between the performance with the internal test set (Spain) and external test set (Brazil), although, overall, the results are reliable in both scenarios. For instance, the main differences between the two tests were in the positive and negative predictive values (PPV and NPV), where the external tests had higher PPV, but lower NPV than the internal tests for depression and anxiety. Despite these differences, the balanced accuracy, sensitivity, and specificity values were consistent for all models across the internal and external tests for depression and anxiety.

**Table 3:** Performance metrics presented based on the results of 3 different models. Results for Spain represent the internal test set results, while those for Brazil are the external set results

	Spain Test			Brazil Test		
	
Metric	Elastic net	RF	XGB	Elastic net	RF	XGB
Depression						
Balanced accuracy	0.79	0.78	0.77	0.76	0.76	0.76
Sensitivity	0.78	0.74	0.74	0.74	0.74	0.75
Specificity	0.79	0.81	0.80	0.78	0.79	0.77
PPV	0.49	0.51	0.49	0.78	0.79	0.77
NPV	0.93	0.92	0.92	0.74	0.74	0.74
Anxiety						
Balanced accuracy	0.78	0.78	0.77	0.75	0.74	0.75
Sensitivity	0.78	0.78	0.77	0.73	0.78	0.74
Specificity	0.79	0.78	0.78	0.77	0.71	0.77
PPV	0.49	0.48	0.48	0.76	0.73	0.76
NPV	0.93	0.93	0.93	0.74	0.76	0.75

RF = random forest; XGB = XGBoost; PPV = positive predictive value; NPV = negative predictive value.

**Figure 1:**
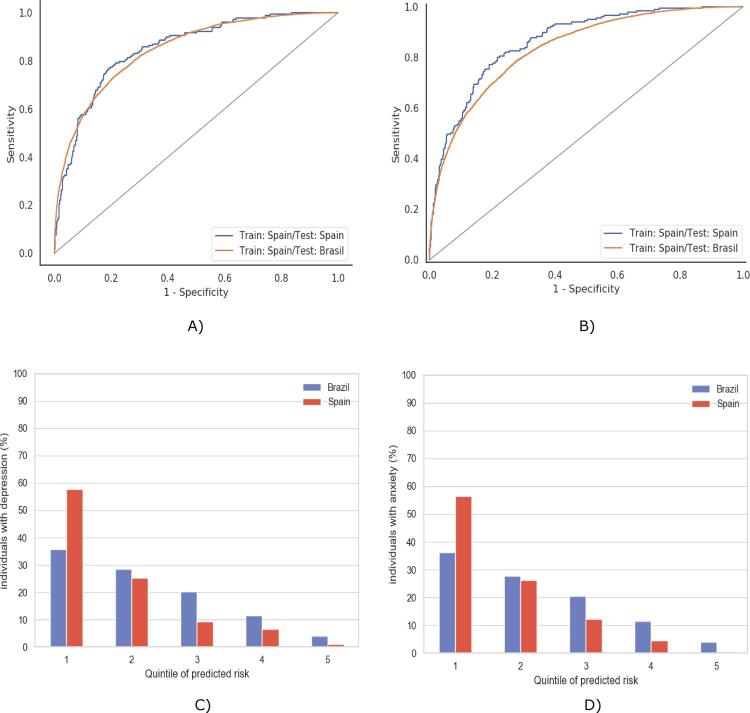
Results for depression and anxiety by training the model with the Spain sample and testing it on the Spain internal set and the Brazil external set: A) ROC curves for the internal (AUC-ROC = 0.85) and external (AUC-ROC = 0.85) tests for presence of depression; B) ROC curves for internal (AUC-ROC = 0.86) and external (AUC-ROC = 0.84) tests for presence of anxiety; C) Percentage of individuals with symptoms of depression by defined quintiles of predicted risk; D) Percentage of individuals with symptoms of anxiety by defined quintiles of predicted risk.

The elastic net model was used for subsequent analyses because of its effectiveness, interpretability, and simplicity. The elastic net AUC-ROC for depression was 0.85 for both the internal and external test (Figure 1A). Its AUC-ROC values for the anxiety model were 0.86 and 0.84 for the internal and external test, respectively (Figure 1B). In both cases, the curves for the internal and external sets are very similar, with performance for the Spain data being slightly better overall.

The calibration of the model was also analyzed, by evaluating the concentration of the outcome within the percentiles of predictions. In Figures 1C and 1D, significant differences were identified between the countries. In order to generate the image, participants were sorted by their predicted value into 5 defined quintiles of predictions. For each of these groups, the percentage of participants that had the outcome with respect to the total number of individuals with the outcome were assessed. More specifically, almost 60% of the participants with depression (first red bar) are within the 20% highest predictions (Figure 1C). In other words, a high prediction from the model is highly predictive of the outcome. However, a reduction was observed in the Brazilian sample. Notably, around 35% of the participants within the 20% highest predictions have depression. This may indicate signs of prediction bias coming from the Spanish sample. Almost the exact same pattern is observed for anxiety (Figure 1D).

**Figure 2:**
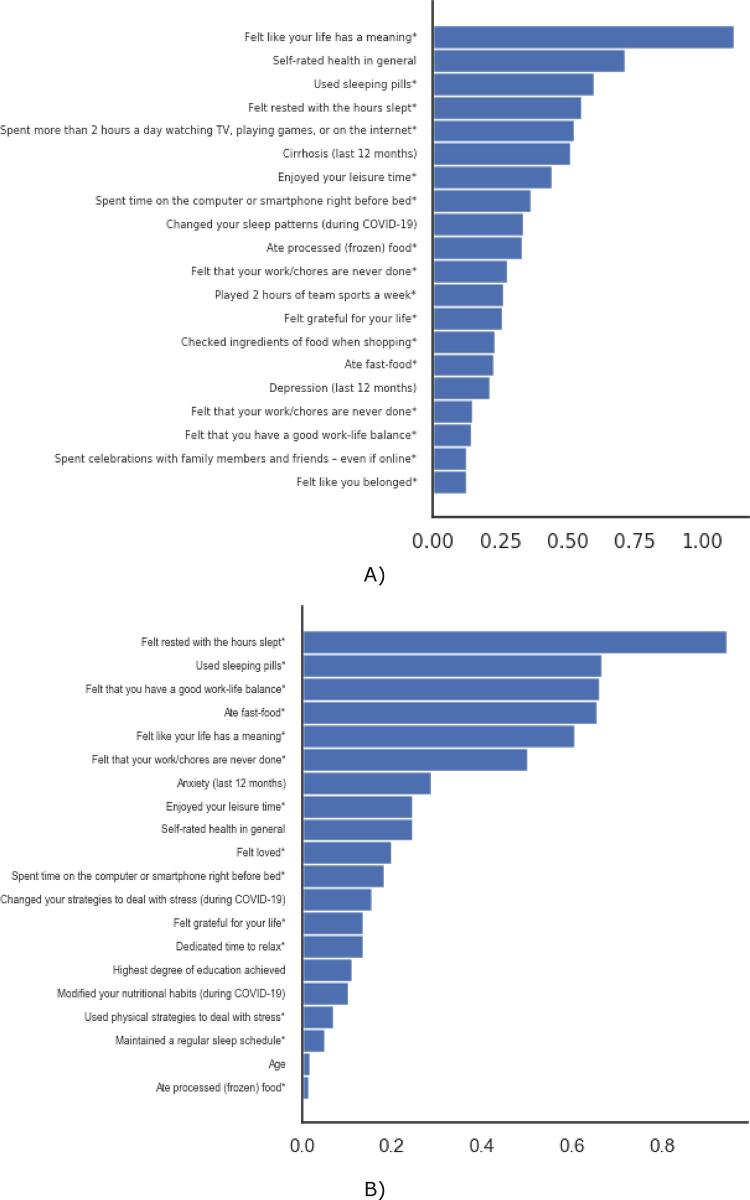
Importance of different variables as predictors of depression and anxiety according to the Spain sample; A) Predictors of depression; B) Predictors of anxiety; * Indicated in the last 30 days.

In addition, the importance of each question on the SMILE scale was examined as an independent lifestyle variable for predicting the presence of depression and anxiety symptoms. Each question was separately evaluated for its projective capacity to suggest presence of depression and presence of anxiety symptoms in the Spanish sample. All questions considered were based on respondents’ daily routines and feelings within the last month. Analysis of the predictors of depression identified the question, “Do you feel that your life has a meaning?” as the most important predictor of the disorder, followed by the question, “How would you rate your health in general?” (from the SRH) and “Do you use sleeping pills?” (Figure 2A). With regards to the predictors of anxiety, the most important SMILE question to predict the presence of the disorder was, “Do you feel rested with the number of hours you sleep?”, followed by “Do you use sleeping pills?” and “Do you feel that you have a good work-life balance?” (Figure 2B).

## Discussion

Findings from this study indicate that variables related to lifestyle, as assessed by the SMILE scale, successfully predicted common mental disorders such as depression and anxiety in a Brazilian sample using a machine learning model developed on a Spanish sample during the early stages of the COVID-19 epidemic. Additionally, the results highlight the importance of different lifestyle factors and general self-concerns as predictors of these disorders, with perception about meaning of life, self-rated health, and use of sleeping pills being the most important predictors of depression, and restful sleep, use of sleeping pills, and perception about work-life balance being the most important predictors of anxiety.

The COVID-19 epidemic started at different times in Spain and Brazil, where the population of Spain was affected by the first wave of the contagious virus prior to escalation of the outbreak in the Brazilian population. By assessing lifestyle behaviors in the early stages of the epidemic in Spain using a multidimensional lifestyle questionnaire (SMILE), we developed predictive models with three different machine learning methods using a subset of the Spanish sample. The models were then tested internally (with the remaining subset of the Spanish sample) and later tested externally with the full Brazilian sample. The consistency in performance metrics between the different methods, as well as the similarities between the internal and external tests suggest that the internally developed models were able to predict the presence of depression and anxiety during the COVID-19 pandemic in the external set, which is the main, novel finding from this research study.

To our knowledge, this is the first study to develop machine learning models in one country that are equally effective in predicting the presence of common mental health disorders in a different country. Implications from this study indicate that elastic net, random forest, and XGBoost are reliable techniques for transnational prediction of common mental disorders such as depression and anxiety. The cross-fertilization of independent analyses carried out in different contexts is a much-desired goal of contemporary epistemology, decision science, and computer science, among other fields of knowledge. Prior literature has found many similarities in lifestyle factors that correlate to presence of mental disorders such as depression and anxiety in underdeveloped, developing, and developed countries.^23,24^ Recent studies conducted during the COVID-19 outbreak have also indicated higher prevalence of depression and anxiety symptoms in the general population during this period.^25,26^ Considering the severe consequences of the pandemic in highly affected countries such as Brazil and Spain, a recent study conducted in these regions found an association between unhealthy lifestyle changes and presence of depression and anxiety among essential workers during the COVID-19 pandemic.^16^ With this in mind, taking into consideration the similarities across different countries in lifestyle adversities due to the COVID-19 crisis, it is possible that the models developed and used in this study may be able to reliably capture predictive factors of the presence of common mental disorders in different regions of the world.

We also assessed the importance of each lifestyle variable on the SMILE scale as independent factors associated with presence of depression and anxiety using machine learning methods. We used the Spain training sample to evaluate the importance of each independent question in predicting presence of the mental disorders of interest. Intriguingly, we found the answers to the question, “Do you feel that your life has a meaning?” to be the predictor most associated with presence of depression. Prior studies have reported an inverse relationship between self-perceived meaning of life and symptoms of depression, concurring with the current findings of our study.^27,28^ However, we believe this to be the first study indicating that perception of meaning of life carries greater significance in predicting symptoms of depression than other lifestyle factors. The high importance factor seen in this variable could be due to the cumulative contribution of many different aspects of lifestyle towards a sense that life is meaningless during the COVID-19 pandemic. Self-rated health was the second most important predictor of presence of depression in this sample during the pandemic, with prior literature also suggesting a strong association between subjective well-being and symptoms of depression.^29,30^ Among the rest of the factors, sleep medications and sleep quality were found to be among the strongest predictors of depression, which has also been suggested in previous findings.^31,32^

Furthermore, we assessed the importance of each lifestyle variable on the SMILE scale as independent predictors of anxiety in the Spanish training sample. The leading, most significant predictor of presence of anxiety was the answer to the question, “In the last month, how often do you feel rested with the number of hours you sleep?”. Similarly, the second most important predictor was related to daily use of sleeping medications during the COVID-19 pandemic. It is known that sleep disturbances and anxiety are bidirectionally associated,^33^ and this association was expectedly evident during the early stages of the pandemic.^34^ Stressful life events, such as the COVID-19 pandemic, which threaten one’s psychological and physical well-being are likely to cause increased sleep disturbances in the population.^35^ Indeed, sleep problems have been highly prevalent during COVID-19, with approximately 40% of the general population reporting poor sleep quality in the early stages of the pandemic.^36^ The confinement period during the pandemic has led to changes in social and environmental cues important for circadian rhythms and the sleep-wake cycle, including the lack of fixed schedules for working, eating, exercising, socializing, and similar daily routines.^35^ Changes in the sleep-wake cycle can result in desynchronization between the circadian rhythm and important immune functions, which can affect a person’s physical and mental well-being.^35,37^ In particular, reduced sleep quality was associated with higher levels of depression and anxiety symptoms early on during the COVID-19 lockdown in Italy.^38^ Similarly, our findings indicate that sleep disturbances may be the most important factor in predicting presence of anxiety in the general population in different countries during the COVID-19 pandemic. Additionally, perception of work-life balance, meaning of life, and consumption of fast food were other variables from the SMILE scale related to the presence of anxiety, corresponding with previous studies indicating associations between these variables and anxiety.^39-41^

Findings from our study could have essential clinical implications for clinical professionals and researchers around the world. Considering the effectiveness of our models across different countries, the method developed could be adapted and used across a vast number of countries, especially when countries are in similar situations such as during the global COVID-19 pandemic. In addition, to our knowledge, this is the first study using machine learning methods to predict which lifestyle behaviors are able to accurately indicate positive screening for depression and anxiety, two of the most common mental disorders globally. Observing the most prevalent lifestyle behaviors among people with depression and anxiety using this model could be the first step towards creating a protocol of targeted interventions addressing unhealthy lifestyle behaviors that increase the likelihood of presence of these symptoms, such as sleep hygiene.

The main limitation of our study is the cross-sectional design. Considering that the lifestyle behaviors (predictors) and the presence of depression/anxiety symptoms (outcomes) were assessed at the same point in time, inference of a causal relationship between the variables is limited. Instead, it depicts a snapshot of a very dynamic process (comprising both the dynamics of the epidemic itself and how people cope with the challenges it poses over time). Furthermore, it is important to highlight that presence of anxiety and depression was assessed using a screening test, and replication of this data assessing them using a structured, albeit much more labor-intensive, clinical interview is encouraged. There are also limitations regarding the lack of fine-tuning of the model for the Brazilian sample; although we showed that the model performs similarly in both countries, the model was not further trained on a subset of Brazilian data, which could potentially improve the model for that sample. In contrast, a major advantage of this study is the consistency in performance metrics across three highly reliable machine learning methods for development of predictive models, which also achieved high AUC-ROC values for the internal and external tests. In addition, another major advantage is that the trained sample of the model was from Spain, one of the first countries affected by the pandemic, which was then tested in Brazil, where the epidemic started at a later period. This is an encouraging sign for development of prevention guides using this model. Lastly, the findings from this paper are in accordance with previous research indicating important mental health burdens related to lifestyle behaviors, especially during the COVID-19 pandemic.

Implications from this study could be highly significant in the approach towards developing targeted approaches to promote healthy lifestyles that might help reducing the burden of common mental disorders.
